# Practice recommendations regarding parental presence in NICUs during pandemics caused by respiratory pathogens like COVID-19

**DOI:** 10.3389/fped.2024.1390209

**Published:** 2024-06-13

**Authors:** Marsha Campbell-Yeo, Fabiana Bacchini, Lynsey Alcock, Souvik Mitra, Morgan MacNeil, Amy Mireault, Marc Beltempo, Tanya Bishop, Douglas M. Campbell, Addie Chilcott, Jeannette L. Comeau, Justine Dol, Amy Grant, Jonathon Gubbay, Brianna Hughes, Amos Hundert, Darlene Inglis, Alanna Lakoff, Yasmin Lalani, Thuy Mai Luu, Jenna Morton, Michael Narvey, Karel O’Brien, Paula Robeson, Michelle Science, Prakesh Shah, Leah Whitehead

**Affiliations:** ^1^ Faculty of Health, School of Nursing, Dalhousie University, Halifax, NS, Canada; ^2^ Department of Pediatrics, IWK Health, Halifax, NS, Canada; ^3^ Canadian Premature Babies Foundation, Toronto, ON, Canada; ^4^ Department of Pediatrics, University of British Columbia, Vancouver, BC, Canada; ^5^ Montreal Children’s Hospital, McGill University Health Centre, Montreal, QC, Canada; ^6^ Department of Pediatrics, St. Michael’s Hospital, Toronto, ON, Canada; ^7^ Niagara Region Public Health, Niagara, ON, Canada; ^8^ Faculty of Medicine, Dalhousie University, Halifax, NS, Canada; ^9^ Maritime SPOR SUPPORT Unit, Nova Scotia Health, Halifax, NS, Canada; ^10^ Department of Laboratory Medicine & Pathobiology, University of Toronto, Toronto, ON, Canada; ^11^ Department of Nursing, University of Prince Edward Island, Charlottetown, PE, Canada; ^12^ Loyalist College, Belleville, ON, Canada; ^13^ Humber River Health, Toronto, ON, Canada; ^14^ Département de Pédiatrie, Centre Hospitalier Universitaire Sainte-Justine, Montréal, QC, Canada; ^15^ Pickle Planet, Moncton, NB, Canada; ^16^ Department of Pediatrics, Children’s Hospital Research Institute of Manitoba, Winnipeg, MB, Canada; ^17^ Department of Pediatrics, Mount Sinai Hospital, Toronto, ON, Canada; ^18^ Department of Department of Paediatrics, University of Toronto, Toronto, ON, Canada; ^19^ Children’s Healthcare Canada, Ottawa, ON, Canada; ^20^ Department of Paediatrics, SickKids Hospital, Toronto, ON, Canada

**Keywords:** COVID-19, neonatal care, parental presence, practice recommendations, participatory research (PR), pandemic planning, respiratory pathogens

## Abstract

**Aim:**

To co-create parental presence practice recommendations across Canadian NICUs during pandemics caused by respiratory pathogens such as COVID-19.

**Methods:**

Recommendations were developed through evidence, context, Delphi and Values and Preferences methods. For Delphi 1 and 2, participants rated 50 items and 20 items respectively on a scale from 1 (very low importance) to 5 (very high). To determine consensus, evidence and context of benefits and harms were presented and discussed within the Values and Preference framework for the top-ranked items. An agreement of 80% or more was deemed consensus.

**Results:**

After two Delphi rounds (*n* = 59 participants), 13 recommendations with the highest rated importance were identified. Consensus recommendations included 6 *strong* recommendations (parents as essential caregivers, providing skin-to-skin contact, direct or mothers' own expressed milk feeding, attending medical rounds, mental health and psychosocial services access, and inclusion of parent partners in pandemic response planning) and 7 *conditional* recommendations (providing hands-on care tasks, providing touch, two parents present at the same time, food and drink access, use of communication devices, and *in-person* access to medical rounds and mental health and psychosocial services).

**Conclusion:**

These recommendations can guide institutions in developing strategies for parental presence during pandemics caused by respiratory pathogens like COVID-19

## 1 Introduction

Nearly 400,000 babies are born in Canada each year. Of these, approximately 15,000 babies are admitted to tertiary neonatal intensive care units (NICUs) annually (1). About 8% will be born preterm (<37 weeks gestational age), with many requiring neonatal intensive care (1). Infants born extremely preterm are the most vulnerable, but even those delivered one to two weeks early can face immediate and long-term challenges, including developmental delays, and social and behavioural problems (2).

Despite significant improvement in survival rates of preterm infants over the past decade, there has been less improvement in associated morbidity and need for additional health care or neurodevelopmental supports (3). Emphasis on greater parental presence, with parents being designated as essential caregivers in the NICU, has shown to be a beneficial component of care (2, 4). Greater parental presence has also been associated with improved infant growth and developmental outcomes, faster time to reach full enteral feeding, greater provision of mother's own breastmilk (MOM), and reduced sepsis, medical procedures, pain response, physiologic stress, duration of hospital stay, and major morbidities (5).

The pandemic has heightened social inequities and mental health concerns (6). While there was some variation on parent presence policies, primarily regarding siblings, extended family or support people, before the COVID-19 pandemic, 24/7 parent presence was the accepted practice in most Canadian NICU's. Parents of infants requiring neonatal care report higher levels of immediate and posttraumatic stress, anxiety, depression, and adverse parenting outcomes than parents of healthy newborns, all of which have been worsened during the COVID-19 pandemic (7, 8).

With the aim to reduce viral spread, policymakers had to rapidly institute public health policies, often with limited information and engagement with families, patients or other relevant partners. Most hospitals restricted access, many leaving patients, including infants, without a support person or parent, even during extreme illness or death (8). At the pandemic's onset, most Canadian NICUs imposed varying degrees of parental presence restrictions, even within similar viral spread communities (9). Presently, there is continued variation in parental presence policies across Canadian NICUs, with some reverting to pre-COVID policies and others maintaining restrictions on parent and family access.

These restrictive COVID-19 policies have been associated with adverse infant health and parent mental health outcomes, reduced family-centered care delivery, and parent access to education, support, and physical needs (7, 10). Globally, NICUs' pandemic response led to significant policy changes, negatively affecting breastfeeding, parental bonding, caregiving involvement, parental mental health, and staff stress (4).

The aim of this study was to co-create Canadian consensus practice recommendations regarding physical parental presence in NICUs during pandemics caused by respiratory pathogens such as COVID-19.

## 2 Methods

### 2.1 Study design and population

The recommendation development process was guided using the AGREE II tool (11). Eligible participants included parents of NICU infants, neonatal healthcare providers and managers, and infectious disease, public or child health care providers or policy makers. The study was approved by the IWK Health Research Ethics Board #1025748, and all participants provided informed consent.

The study was conducted between April 2022 and July 2023 and was carried out in two phases. For phase 1, the Delphi (12) method was used to determine priority recommendations of importance to participants, using two rounds of surveys. The aim was to identify the top 10–12 recommendations. This number, while arbitrary is in keeping with many practice recommendations, and reflects a broad but manageable number to aid in practice uptake and implementation (13). For phase 2, the consensus team was guided by best evidence synthesis, national context, and values and preferences to reach a consensus for the recommendations (14).

### 2.2 Recruitment

A purposive sampling method was used to help facilitate a diverse and cross sectoral representation (e.g., geographic location, parents and multidisciplinary care providers, leaders and policymakers) for all phases of the study.

For phase 1, we aimed to include a minimum of 20–40 participants across relevant groups: parents of NICU infants, multi-disciplinary healthcare providers with expertise in neonatal care, infectious disease, and public health, health system leaders, and policy makers. To recruit participants, the Delphi surveys were disseminated electronically via email or social media channels across the study teams networks including the Canadian Premature Babies Foundation (CPBF), Canadian Neonatal Network (CNN), and Children's Healthcare Canada. Surveys were accessed on the Research Electronic Data Capture (REDCap) platform hosted at IWK Health, located in Eastern Canada (15). Surveys were open until our goal sample was reached.

For phase 2, we aimed to include a purposive sample of a minimum of 20 participants.

### 2.3 Data collection

The Delphi surveys were co-created by the research team based on previously circulated national neonatal survey data (parents, health care providers and leaders) and available peer-reviewed published data on the most reported practice gaps, consequences, or impact of the COVID-19 pandemic on families of infants requiring care in a NICU (9, 16–18).

The first Delphi included 50 possible recommendation items to be included as national recommendations (Supplementary Materials). Participants were asked to rate each item on their perceived importance on a Likert scale from 1 (very low importance) to 5 (very high importance). A composite score for each item was calculated based on the sum of the mode, median, and mean. Any item with a mode, median, or mean of less than “4” on the 5-point Likert scale (i.e., rated less than “high” or “very high” importance), along with any interquartile range of more than 1-point difference, was removed from the next round because they were not of high importance.

The top 20 items with the highest composite score proceeded to the second round of the Delphi (Table 1). During the second Delphi, participants were asked to rank each item in order of importance from greatest (1) to least (20) impact on safety, parental and healthcare provider mental health, and infant health outcomes. Items were reverse scored (i.e., a rank of 1 was scored as a 20) and scores were summed for each question across respondents to calculate priority scores. The top-rated items with the highest priority scores were items moved forward to the next stage of the study to be considered for the national consensus recommendations.

**Table 1:** Delphi 1, rating items of importance for consensus discussion.

Item topic	Composite score^a^	Median (IQR)^b^
Access for parents to participate in usual care tasks for their infant	14.9	5 (0)
Access for parents to provide skin-to-skin contact (as often as they want or for a specific amount of time [in hours).	14.9	5 (0)
Access to touch infant in the incubator/cot	14.9	5 (0)
Access for mothers to breastfeed/access to lactation support and breast pump	14.9	5 (0)
Number of infant parents able to be with the infant in the NICU at the same time	14.8	5 (0.3)
Access and support to participate in daily medical rounds	14.7	5 (0)
Provision of PPE (i.e., face masks) and hand sanitizer	14.6	5 (1.0)
Access to parent/peer-to-peer support	14.6	5 (1.0)
Access to toilet/shower facilities	14.5	5 (1.0)
Provision of allocated space to sleep	14.4	5 (1.0)
COVID-19 screening to enter the NICU	14.4	5 (1.0)
Use of parents’ own technology devices (e.g., phone, tablet, etc.) in the NICU	14.3	5 (1.0)
Positive COVID-19 cases of parents/family members/support people	14.3	5 (1.0)
Access to therapy services and psychological and emotional supports	14.3	5 (1.0)
Provision of information on infection risk (including risk-reducing behaviours, safe use of PPE) to make informed decisions for safety in the NICU	13.7	4.5 (1.0)
Inclusion of parent partners in designing infection control/pandemic response planning/parent related NICU policies	13.3	4 (1.0)
Number of infant (non-parent) primary care provider/guardians able to be with the infant in the NICU	13.2	4 (1.0)
Instruction on expectation of wearing a face mask (e.g., upon admission to hospital, in NICU always, only when not physically distancing	13.0	4 (1.0)
Access to current policy procedure, recent policy updates, and explanation of policy necessity for parent(s)	12.3	4 (1.0)
Access to allocated space to eat and drink (in family lounge, in staff cafeteria, at infants’ bedside)	12.3	4 (1.0)

^a^ Sum of Median, Mean, Mode. Minimum value of 3, maximum value of 15 (19).

^b^ Score ranges from 1 to 5, 1 being least important and 5 being the most important item for discussion of inclusion in national recommendations.

Survey data were collected through REDCap, a secure web application for building and managing online research surveys and databases, which can be accessed through a shareable public web address (15). Data were collected up to the point that participants completed the survey. If participants chose to withdraw before the survey ended, REDCap recorded the data that the participant completed up until the point of withdrawal.

For phase 2, a rapid synthesis of the evidence related to the benefits and harms associated with each of the top ranked items was presented to the consensus panel. See Supplementary Material 1 for search strategy. The rapid review was conducted to accelerate the synthesis process by streamlining the systematic review methods via limiting the search to published literature, including English articles only and having one person screen and abstract the data and another verify (20). Consensus was established when threshold for agreement was met, if voting was equal to or exceeding 80% agreement (21).

A decision aid summarizing the evidence was presented for each item, which included the certainty of evidence for each outcome. Items were discussed, considering context, values and preferences. Panel participants were then asked to vote, yes or no, via an anonymous online portal regarding the recommendation of each of the top ranked items.

Recommendations for each item were either “strong” or “conditional” based upon the certainty of evidence for the outcomes. A recommendation was deemed “strong” if the consensus panel was confident that the desirable effects of adherence to a recommendation outweighed the undesirable effects and that given the certainty of the evidence and consistency of values and preference, it would be unlikely that the recommendation would change with new evidence (21). A recommendation was deemed “conditional” if there was a small margin between favorable and unfavorable outcomes, the consensus panel concluded that the desirable effects of adherence to the recommendation probably outweighed the undesirable effects or if, the evidence was of lower quality, there was greater variability in individual values and preferences and there was a possibility that the recommendation may change with new evidence (21). Recommendations were based on current COVID data regarding virulence of covid-19 and associated low risk for newborns.

### 2.4 Analysis

Categorical data are expressed as frequencies and percentages. Continuous data are expressed as means and standard deviations (SD) for parametric data, and median and interquartile range (IQR) for nonparametric data. The median was used as it is the recommended measure for Likert scales (19). The IQR was applied, as it considered the spread of responses around the median to gauge the level of agreement when establishing consensus. To discern the hierarchy of items, a composite score, incorporating the mean, median, and mode, was computed (19).

Following a rapid synthesis of evidence, certainty of evidence was determined using the GRADE (Grading of Recommendations Assessment, Development and Evaluation) framework (21). In keeping with this framework, certainty of evidence was determined from, risk of bias, inconsistency, indirectness, imprecision, and publication bias (50).

The methodological quality of systematic reviews including clinical trials was determined using AMSTAR (Assessment of Multiple Systematic Reviews) (22). For instances where little to no quantitative data existed, narrative summaries of qualitative data were provided, and the quality of the data was determined using the Mixed Methods Appraisal Tool (MMAT) (23).

## 3 Results

The demographic information of the Delphi participants (*n* = 59) and consensus panel members can be found in Table 2. The national consensus panel consisted of 21 participants including: parents of NICU babies; health care providers (neonatology, pediatrics, infectious disease, public health), health system leaders, and policy makers (Table 2). Results from the first and second Delphi surveys can be found in Tables 1, 3 respectively. National recommendations were established through discussion at 13 consensus panel meetings held between October 2022 and July 2023. An example of evidence presented to the consensus panel to support the benefits and harms associated with each recommendation can be found in Tables 4, 5. Certain items of importance from the second Delphi (Table 3) were combined, divided, added, or removed after discussions with the panel. Given the current evidence available in the literature as well as the values and preferences of members of our consensus panel, our final number of recommendations totaled 13. We aimed to establish a maximum of 10–12 national recommendations, to facilitate practice uptake (42). Of the 13 recommendations, 6 were strong recommendations in favour with 100% consensus, and 7 were conditional recommendations in favour with consensus ranging from 84%–100% (Table 6 and for a visual summary, Supplementary Material 2).

**Table 2:** Participant demographics.

Characteristic	Delphi 1 *N* = 39	Delphi 2 *N* = 20	Consensus panel *N* = 21
Type of participant, *n* (%)			
Parent of infant requiring NICU care	19 (49)	10 (50)	5 (25)
Mother, *n* (%)^a^	17 (90)	9 (90)	5 (100)
NICU healthcare provider	14 (36)	8 (40)	13 (70)
Physician, *n* (%)^a^	5 (36)	4 (50)	9 (69)
Registered Nurse, *n* (%)^a^	5 (36)	3 (38)	3 (23)
Neonatal nurse practitioner, *n* (%)^a^	2 (14)	1 (12)	1 (8)
Pharmacist, *n* (%)^a^	1 (7)	0 (0)	0 (0)
Dietitian, *n* (%)^a^	1 (7)	0 (0)	0 (0)
Leader/Policy/Knowledge mobilization	4 (10)	1 (5)	1 (5)
Researcher	2 (5)	1 (5)	2 (10)
Geographic location: Canadian province, *n* (%)			
Alberta	2 (5)	1 (5)	0 (0)
British Columbia	3 (8)	2 (10)	1 (5)
Manitoba	5 (13)	0 (0)	1 (5)
Newfoundland and Labrador	1 (3)	0 (0)	0 (0)
New Brunswick	0 (0)	0 (0)	1 (5)
Nova Scotia	4 (10)	4 (20)	6 (30)
Ontario	19 (49)	11 (55)	9 (45)
Prince Edward Island	1 (3)	0 (0)	0 (0)
Quebec	4 (10)	2 (10)	2 (10)

Percentages rounded to the nearest tenth.

^a^ % of subgroup total.

**Table 3:** Delphi 2, ranking items of importance for consensus discussion*.*

Item topic	Median (IQR)^a^
Access for mothers to breastfeed, breastfeeding encouragement, breast pumps, and lactation support	19.0 (2.5)
Access for family members/support people to provide SSC	18.5 (2.75)
Access to touch infant in the incubator/cot	18.0 (2.5)
Access for parents to participate in usual care tasks for their infant	17.0 (4)
Access and support to participate in daily medical rounds (virtually, in-person at bedside, in-person away from bedside)	15.5 (5.75)
Number of infant parents and (non-parent) caregiver/guardians able to be with the infant in the NICU at the same time	14.5 (7.75)
Policy for positive COVID-19 birthing person, and presence of positive COVID-19 parents/caregivers/family members/support people	11.0 (6)
Inclusion of parent partners in designing infection control/pandemic response planning/parent related NICU policies	11.0 (9)
Access to parent/peer-to-peer support	11.0 (7.5)
Access to allocated space to eat and drink (in family lounge, in staff cafeteria, at infants’ bedside)	10.0 (4)
Access to therapy services and psychological and emotional supports	9.0 (6.5)
Use of parents’ own technology devices (e.g., phone, tablet, etc.) in the NICU	6.0 (10)

^a^ Median score of items from 1 to 20, 1 being least important and 20 being the most important item for discussion of inclusion in national recommendations.

**Table 4:** Anticipated benefits of skin-to-skin contact (SSC).

Quality assessment							No. of participants		Effect	Certainty of evidence (GRADE)	Quality of review (AMSTAR)	Reference
No. of studies	Study design	Risk of bias	Inconsistency	Indirectness	Imprecision	Publication bias	Intervention	Control				
Infant pain												
5	Systematic review (5 RCT)	Not serious	Not serious	Not serious	Serious	Not serious	SSC during heel lance (*n *= 129)	Control (swaddling or no treatment) during heel lance (*n* = 138)	Significant effect in favour of SSC MD −3.21 (−3.94, −2.47	Moderate ⊕ÅÅO	High	(24)
Parent mental health												
4	Systematic review (3 RCT, 1 Non-randomized)	Serious	Serious	Not serious	Serious	Not serious	SSC 767	–	SSC associated with 1.04% reduction in standardized depression scores SMD −1.04 (−1.30, −0.79)	Very low ⊕OOO	Moderate	(25)
Infant mortality												
8	Systematic review (8 RCT)	Not serious	Not serious	Not serious	Serious	Not serious	SSC 888	Conventional neonatal care 848	60% reduction in risk of infant mortality at discharge or 40–41 weeks’ PMA with SSC MD 0.60 (0.39, 0.92)	Moderate ⊕ÅÅO	High	(26)
Infant development												
1	Systematic review (1 RCT)	Not serious	Serious	Not serious	Serious	Not serious	SSC 308	Conventional neonatal care 280	20% relative reduction in cerebral palsy, deafness, and visual impairments at 12 months’ corrected age MD 0.80 (0.50, 1.29)	Low ⊕⊕OO	High	(26)
1	Systematic review (1 RCT)	Not serious	Serious	Not serious	Serious	Not serious	SSC 308	Conventional neonatal care 271	Mean Griffith quotient for psychomotor development at 12 months’ corrected age in the intervention groups was 1.05 higher MD 1.05 (−0.75, 2.85)	Low ⊕⊕OO	High	(26)
Infant physiologic stability												
15	Systematic review (1 RCT, 1 Randomized Crossover, 12 Pre-Post, 1 Intervention)	Not serious	Serious	Not serious	Serious	Not serious	SSC 612	–	Heart rate lower in intervention group WMD −0.41 (−2.25, 1.42)	Low ⊕⊕OO	Moderate	(27)
12	Systematic review (2 RCT, 1 Randomized Crossover, 9 Pre-Post)	Not serious	Serious	Not serious	Serious	Not serious	SSC 564	–	Respiratory rate lower in intervention group WMD −3.17 (−5.15, −1.19)	Low ⊕⊕OO	Moderate	(27)
14	Systematic review (3 RCT, 1 Randomized, 10 Pre-Post)	Not serious	Serious	Not serious	Serious	Not serious	SSC 675	–	Oxygen saturation higher in intervention group WMD 0.90 (0.35, 1.45)	Low ⊕⊕OO	Moderate	(27)
14	Systematic review (2 RCT, 1 Randomized Crossover, 1 Crossover, 10 Pre-Post)	Not serious	Serious	Not serious	Serious	Not serious	SSC 642	–	Temperature higher in intervention group WMD 0.24 (0.15, 0.33)	Low ⊕⊕OO	Moderate	(27)
Breastfeeding												
6	Systematic review (6 RCT)	Not serious	Not serious	Not serious	Serious	Not serious	762	691	SSC associated with an increase in the likelihood of exclusive breastfeeding at discharge or 40–41 weeks’ PMA MD 1.16 (1.07, 1.25)	Moderate ⊕ÅÅO	High	(26)
8	Systematic review (6 RCT, 1 Observational, 1 Case control)	Not serious	Serious	Not serious	Not serious	Not serious	605	457	SSC increases the likelihood of exclusive breastfeeding by 39% at 1–4-month follow-up MD 1.39 (1.11, 1.74)	Moderate ⊕ÅÅO	Moderate	(27)

**Table 5:** Anticipated Harms of skin-to-skin contact.

Quality assessment							No. of participants		Effect	Certainty of evidence (GRADE)	Quality of review (AMSTAR or MMAT)	References
No. of studies	Study design	Risk of bias	Inconsistency	Indirectness	Imprecision	Publication bias	Intervention	Control				
HCP infection rate												
1	Observational	Not serious	Not serious	Not serious	Serious	Not serious	2,884	–	Presence of parents does not affect infection rates among HCPs 1 (0.69, 1.06)	Very low ⊕OOO	Moderate (MMAT)	(28)
Infant COVID+												
6	4 observational, 1 retrospective cohort, 1 case control	Not serious	Not serious	Not serious	Serious	Not serious	652	–	Incidence of infants who test COVID+ and had SSC, rooming-in, and/or breastfeeding with COVID+ parent 2.3 (1.1, 3.4)	Very low ⊕OOO	Moderate (MMAT)	(29–33)
13	5 observational, 2 retrospective cohort, 1 case control, 1 case series, 2 cohort, 2 review	Not serious	Not serious	Not serious	Serious	Not serious	2,546	–	Incidence of infants who test COVID+ and have COVID+ parent 5.6 (4.7, 6.5)	Very low ⊕OOO	Moderate (AMSTAR, MMAT)	(29–41)
Infant COVID symptoms												
4	3 observational, 1 review	Not serious	Not serious	Not serious	Serious	Not serious	1,035	–	Incidence of infants who have a COVID+ parent and COVID symptoms 1.9 (1.1, 2.8)	Very low ⊕OOO	Moderate (MMAT, AMSTAR)	(29, 32, 34, 39)
3	2 observational, 1 review	Not serious	Not serious	Not serious	Serious	Not serious	26	–	Incidence of infants who test COVID+ and have COVID symptoms 38.5 (19.8, 57.2)	Very low ⊕OOO	Moderate (MMAT, AMSTAR)	(32, 38, 40)
Infant mortality												
2	1 Observational, 1 cohort	Not serious	Not serious	Not serious	Serious	Not serious	624	–	Incidence of infant mortality in NICU (global) when parent is COVID+ 1.4 (0.5, 2.4)	Very Low ⊕OOO	High (MMAT)	(38, 41)

**Table 6:** Consensus recommendations*.*

Item topic	Votes, Yes^a^ n (%)	Votes, No^a^ n (%)	Consensus^b^ (%)
Parent(s)^c^ should be considered essential caregivers for their infant in the NICU. Strong recommendation in favour.	17 (85)	0 (0)	100
Parent(s) should have *unrestricted*^d^ access to breastfeed and to receive breastfeeding supports (including early hand expression, pumping and pumps, encouragement, and lactation support) for their infant in the NICU. Strong recommendation in favour.	18 (90)	0 (0)	100
Parent (s) should have *unrestricted* access to provide skin-to-skin contact for their infant in the NICU. Strong recommendation in favour.	20 (100)	0 (0)	100
NICU parent partners/stakeholders should be included in co-designing/decision-making for parent related NICU policies (e.g., infection control, response planning. Strong recommendation in favour.	19 (95)	0 (0)	100
Parent(s) should have *uninterrupted*^e^ access to mental health and psychosocial support services while their infant is admitted to the NICU. Strong recommendation in favour.	19 (95)	0 (0)	100
Parent(s) should have *uninterrupted* access to attend medical rounds while their infant is admitted to the NICU. Strong recommendation in favour.	18 (90)	0 (0)	100
Parent(s) should have *unrestricted, in-person* access to attend medical rounds while their infant is admitted to the NICU. Virtual care services may be preferred, based on the local context or if parent need/parent preference warrants it. Conditional recommendation in favour.	18 (90)	0 (0)	100
Parent(s) should have *unrestricted in-person* access to mental health and psychosocial support services while their infant is admitted to the NICU. Virtual care services may be preferred, based on the local context or if parent need/parent preference warrants it. Conditional recommendation in favour.	16 (80)	3 (15)	84
Parent(s) should have *unrestricted* access to provide hands-on care tasks for their infant in the NICU. Conditional recommendation in favour.	19 (95)	0 (0)	100
Parent(s) should have *unrestricted* access to provide healing touch for their infant in the NICU. Conditional recommendation in favour.	18 (90)	0 (0)	100
Parent(s) should have *unrestricted* access to food and allocated spaces to eat/drink while their infant is admitted to the NICU. Conditional recommendation in favour.	18 (90)	0 (0)	100
*Two* parent(s) have *uninterrupted* access to be present while their infant is admitted to the NICU. Conditional recommendation in favour.	17 (85)	1 (5)	94
Parent(s) should have *unrestricted* access to use communication devices (their own or hospital devices) for remote connectedness and support (with partners, family, peers, etc.) while they are in the NICU with their infant. Conditional recommendation in favour.	15 (75)	2 (10)	88

^a^ Data are shown as No. (%) of total participants, *N* = 21. Variations in the total # of votes reflect that some members were unable to attend all meetings.

^b^ Data are shown as percentage of people who voted “yes” in favour of the recommendation, out of the number of people who voted. Consensus is considered as a minimum of 80% of the votes for “yes”.

^c^ *Parents* were considered biological mothers/fathers, non-biological mothers/fathers and is inclusive of all sex and gender parent coupling.

^d^ *Unrestricted* was considered as no restrictions-two parent/caregiver(s) in NICU at the same time, amount of time present in NICU (24/7), movement between home and hospital, symptomatic status or COVID-19 positivity status (requires PPE that is effective and appropriate for the tasks).

^e^ *Uninterrupted* was considered as provision of the same service that was provided prior to pandemic.

During consensus panel meetings, there was considerable discussion surrounding the language used for each recommendation. The term “unrestricted” is used for recommendations that would have no restrictions on (a) two parents in the NICU at the same time, (b) amount of time present in the NICU, (c) movement between home and hospital, (d) COVID-19 symptom status, or (e) COVID-19 positivity status (requires personal protective equipment that is effective and appropriate for the tasks being carried out). The term “uninterrupted” was used to describe provision of services that had been in place before the pandemic (i.e., attendance at medical rounds, mental health support).

## 4 Discussion

The purpose of this study was to co-create national Canadian consensus recommendations to address the unattended negative consequences specific to parental NICU presence and support policies, through engaging stakeholders such as parents, families, healthcare providers, researchers, leaders, and policymakers. These recommendations incorporate best available evidence, context, values, preferences, and priorities for post-COVID-19 recovery, and future pandemics caused by respiratory pathogens that are adaptive and responsive across communities.

To our knowledge, we are the first to propose a set of consensus recommendations that can be applied to the post COVID-19 pandemic recovery and future pandemics caused by respiratory pathogens.

Overall, the panel collectively agreed on the paramount importance of parental access to their babies and the imperative to prevent parent restrictions on parental access in future pandemics. Supported by the evidence, the presence of parents in the NICU demonstrated a substantial positive impact on infant and parent outcomes, outweighing the potential harms or risks associated with being present in the NICU. The first strong recommendation, highlighting that parents are essential caregivers, was added by the consensus panel. This recommendation is imperative for addressing concerns regarding organizational and health system policy formation and the inclusion of families in care practices for post-COVID-19 and future pandemic and other planning (4). Ultimately, this recommendation recognizes parents as essential caregivers, as opposed to visitors in the NICU, and supports that policies be changed to reflect this distinction.

Breastfeeding, provision of MOM and SSC recommendations both had strong, high-quality evidence to support their inclusion as unrestricted in the national recommendations. The COVID-19 pandemic caused widespread decreased in breastfeeding, provision of MOM and SSC, leading to a high risk of negative outcomes for both mothers and their infants (43). Evidence to support breastfeeding and provision of MOM and SSC and their associated immediate and long-term positive outcomes in neonates far outweighed the potential negative effects from COVID-19 (43, 44).

Parent access to psychological support services and to attend medical rounds were each split into two recommendations of “uninterrupted” (strong) and “unrestricted” (conditional). The decision to split these recommendations was made as there was not sufficient evidence in the literature to support that in-person access to these services was significantly more beneficial than virtual access in the context of COVID-19. Parent access to personal support systems using personal communication devices were combined to form one “unrestricted” (conditional) recommendation. This recommendation aligns with previous data reporting wide variation in policies allowing communication devices in NICUs (18).

While the recommendations were co-created using rigorous methods, certain limitations must be acknowledged in this study. Firstly, the phase 1 sample size comprised of only 59 participants which, while adhering to minimum Delphi methods, may limit the generalization across top-ranked items. However, the included items align with findings from larger parent and healthcare provider surveys (9). Secondly, while our findings relate to the Canadian context, the decisions made by the panelists were based on available global evidence on COVID-19 up until the consensus panel meeting. Although re-evaluation may be needed with emerging knowledge in future pandemics, this work provides a robust foundation for guiding current system change and steering future decision-making to reduce future unintended harms during future pandemics across Canada and worldwide. Lastly, the impact of parent, family and care provider restrictions during the pandemic extended beyond the NICU, affecting various care areas, including intensive care, pediatric care, general medical care, hospital care, palliative care, and nursing home care (45). Although our recommendations are tailored to the NICU setting, the framework can be applied to formulate presence recommendations and support policies in these other care contexts.

## 5 Conclusion

Preventing parental presence in the NICU has significant negative effects on parent mental health and well-being, as well as on infant health outcomes. Nationwide consensus in parental presence policies must be achieved to optimize parent and infant health outcomes and ensure equitable provision of neonatal care. Immediate implementation of the recommendations established from this study will reduce unintended harms associated with parent restriction and are useful for current post-recovery and future pandemics caused by respiratory pathogens.

## Data Availability

The original contributions presented in the study are included in the article/**Supplementary Material**, further inquiries can be directed to the corresponding author.
