# Supplementary material for: Practice recommendations regarding parental presence in NICUs during pandemics caused by respiratory pathogens like COVID-19
Source: Front Pediatr. 2024 Jun 13;12:1390209. doi: 10.3389/fped.2024.1390209 (PMC11232356; doi:10.3389/fped.2024.1390209)
Supplement: Supplementary file 1 [file Datasheet1.pdf]

Delphi (Round 1) Survey

WELCOME

You are being invited to participate in consensus building for the research project entitled “Restricted family presence in the NICU during the COVID-19 pandemic: Understanding impact and co-created response (The PRESENCE Study)”. This project is funded through CIHR (Operating Grant), Research Ethics Board Approval #1025748.

The primary goal of this study is to identify the impact of parent presence restriction policies in Canadian NICUs on parents, infants, and healthcare providers and to co-create national recommendations for ongoing pandemic management and post-pandemic policies.

To co-create these national recommendations will take the following steps:

1. Collect evidence of the impact of parent restriction policies in Canadian NICUs through surveys and literature review (ongoing)
2. Create a list of approximately 50 top practice gaps and potential recommendation items, categorized into larger themes (complete)
3. **Stakeholders (you) will rate each category and item according to your opinion on the importance of each category and item for inclusion in national recommendations for pandemic management – This is the current, first round of the Delphi (Round 1) survey that you are being invited to participate in**
4. From the results from the Delphi (Round 1) survey, we will generate a list of top 20 potential recommendation items
5. Stakeholders will rank order each item to form a ranked list of importance of each of the 20 items – This will be the second round of the Delphi (Round 2) survey
6. From the results of the Delphi (Round 2) survey, we will generate a list of top 10 potential recommendation items
7. We will then have a meeting of stakeholders to further prioritize the top 10 potential recommendation items, using techniques including Discrete Choice Experiments and Values and Preferences.
8. We will have a final consensus meeting to discuss the final national recommendations

If you are interested in participating in this Delphi (Round 1) survey, you will be directed to review the consent form and begin the Delphi (Round 1) survey. It should take no longer than 15-20 minutes, and you can return to previous pages at any time.

Are you interested in participating in this Delphi (Round 1) survey?

1, Yes
0, No

INFORMED CONSENT

Research Title
Restricted family presence in the NICU during the COVID-19 pandemic: Understanding impact and co-created response (The PRESENCE Study)

Researchers
Principal Investigators
Marsha Campbell-Yeo, Dalhousie University & IWK Health (Nominated PI)
Fabiana Bacchini, Canadian Premature Babies Foundation

Co-Investigators
Marc Beltempo, McGill University
Jeannette Comeau, IWK Health
Justine Dol, St. Michael's Hospital
Amy Grant, Maritime SPOR SUPPORT Unity
Jonathan Gubbay, Public Health Ontario
Brianna Hughes, Dalhousie University
Amos Hundert, IWK Health
Darlene Inglis, IWK Health
Yasmin Lalani, Canadian Premature Babies Foundation
Thuy Mai Luu, Université de Montréal
Souvik Mitra, IWK Health
Michael Narvey, University of Manitoba
Karel O'Brien, Mount Sinai Hospital
Michelle Science, University of Toronto
Prakeshkumar Shah, Sinai Health System

Funding
CIHR Operating Grant - Emerging COVID-19 Research Gaps and Priorities Funding for the project "Parental presence restrictions due to COVID-19: Examining impact and priorities for neonatal care (The PRESENCE Study)"

Introduction and purpose
You are being invited to participate in co-creating national recommendations about parental presence restrictions during the COVID-19 pandemic across Canadian Neonatal Intensive Care Units (NICU)s.

How will the researchers do the study?
We are asking stakeholders from across Canada to complete an online survey.

What will I be asked to do?
Participating in this study involves completing an online survey (approximately 15-20 minutes). Within the Delphi (Round 1) survey, there are broader categories and individual items within those categories of potential national recommendations for pandemic management and post-pandemic policy related to parental presence across Canadian NICUs. You will be asked to rate each category and individual item (within those categories) on a scale of importance to include in the national practice recommendations,

from 1 (very low importance) to 5 (very high importance). You will also be asked if you wish to continue participating in building national recommendations. This includes the second round of the Delphi (Round 2) survey, and by attending collaborative meetings with a group of stakeholders including parent partners, researchers, hospital leaders, policymakers, and decision-makers.

Potential benefits
There are no direct benefits to you for participation in the study. Indirectly, we will use the results of this study to try to inform a national approach to family presence in the NICU in response to the COVID-19 pandemic, and if necessary, any pandemics in the future, as well as inform a national approach during a post pandemic recovery.

Potential harms and burdens
Participating in this study will not affect your healthcare or job position at your hospital in any way, if applicable. Only the researchers will have access to this data and every effort will be made to ensure that the information you provided remains confidential.

What alternatives to participating do I have?
You do not have to participate in this study. Your participation in this study is entirely voluntary (your choice).

Can I withdraw from the study?
You can withdraw from the study at any time by simply closing the link and no responses will be recorded. We will only see the responses of people who hit "submit". Because the results are anonymous once you have submitted your responses you will not be able to withdraw them from the study. Withdrawal will not affect your healthcare or job position at the hospital in any way, if applicable.

Costs and reimbursements
There are no costs associated with participating in this study. Your answers to the survey will not be linked with your identifying information.

Are there any conflicts of interest?
None of the researchers or site investigators have any conflicts of interest or financial interest in the study results. There are no business interests involved.

What about possible profit from the commercialization of the study results?
There are no possible profits from the commercialization of study results.

How will my privacy be protected?
Information shared while participating in this study will be kept private. Your responses will be assigned an ID number and saved to a file that will not include any identifying information. We will store your email address, if you choose to provide it to take part in future surveys and consensus-building, separately from your survey responses, ensuring that your personal information (email address) is not linked to your responses in any way. All attempts at maintaining confidentiality will be taken. No individualized results will be reported. Responses to the online survey are collected using REDCap survey software and are stored on a protected server at IWK Health. Study documents are stored at the Centre

for Pediatric Pain Research at IWK Health. Only research staff and the IWK Health Research Ethics Board will be able to access study information. Study data will be kept for a minimum of 5 years following publication.

What are my research rights?
If you are interested in participating, please click the 'Submit' button. If you are not interested in participating, simply exit the survey. Completion of the survey indicates that you have agreed to take part in this research and for your responses to be used. In no way does this waive your legal rights or release the investigators, sponsors, or involved in situations from their legal and professional responsibilities. If you have any questions at any time during or after the study about research in general, you may contact the Research Office of IWK Health at (902) 470-7989, Monday to Friday between 8:00am and 4:00pm (AST).

How will I be informed of study results?
A summary of the results will be posted on our website (www.mom-linc.ca) upon study completion.

What if I have study questions or problems?
Please contact Dr. Marsha Campbell-Yeo (marsha.campbell-yeo@dal.ca).

Consent to participate
By clicking "Submit" below on to the survey, you are acknowledging that you have read the letter of information and consent to participate.

[Submit]

BASIC DEMOGRAPHIC INFORMATION

In this section, we will ask you a few basic demographic questions so we can see the general characteristics of what our study participants are like. Please note that your identity will not be connected with any of these answers, and all answers will be kept strictly confidential. No one will be able to identify you (or your infant, if applicable).

Please indicate what province or territory you live in:
1, Alberta
2, British Columbia
3, Manitoba
4, Newfoundland and Labrador
5, New Brunswick
6, Nova Scotia
7, North West Territories
8, Nunavut
9, Ontario
10, Prince Edward Island
11, Quebec
12, Saskatchewan
13, Yukon

Various different stakeholders are participating in this survey. Please indicate if you belong to any of the following stakeholder groups:

1, Parent/primary caregiver/designated guardian of an infant who has been admitted to a NICU any time since March, 2020
2, NICU healthcare provider
3, NICU leadership role (please specify):
4, Executive leadership role (please specify):
5, Other relevant stakeholder (please specify):

IF 1:
What is your relationship to your infant admitted to the NICU?
1, Mother
2, Father
3, Step-mother
4, Step-father
5, Foster mother
6, Foster father
7, Other (please specify below)

> **IF 7:**
> Please specify your relationship to the infant [Textbox]

Please indicate the NICU to which your infant was admitted:
0, Alberta Children's Hospital
1, B.C. Women's & Children's Hospital
2, Cape Breton Regional
3, Centre Hospitalier Universitaire de Sherbrooke
4, Centre Hospitalier Universite Laval
5, Children's Hospital of Eastern Ontario
6, Dr. Everett Chalmers Regional
7, Foothills Hospital (U of Calgary)
8, IWK Health Centre
9, Janeway Children's Health & Rehab Centre
10, Jewish General Hospital
11, Kingston General
12, London Health Sciences Centre
13, McGill University Health Centre-MCH
14, McMaster University Hospital (HHS) Hamilton Health Sciences
15, Moncton Hospital
16, Mount Sinai Hospital
17, Regina General Hospital
18, Royal Alexandra Hospital & University of Alberta Hospital
19, Royal Columbian
20, Royal University Hospital/U of Saskatchewan
21, Royal Victoria Hospital
22, St. Boniface Hospital
23, St. John Regional
24, Stollery Children's Health Centre
25, Sunnybrook Health Sciences Centre
26, Surrey Memorial Hospital
27, The Hospital for Sick Children
28, Université de Montreal Hospital Sainte-Justine
29, Victoria General
30, Windsor Regional Hospital
31, Winnipeg Health Sciences Centre
33, The Ottawa Hospital
32, Other (please specify below)

IF 32:
Please specify the NICU: [Text box]

IF 2:
Which NICU do you work in?
0, Alberta Children's Hospital
1, B.C. Women's & Children's Hospital
2, Cape Breton Regional
3, Centre Hospitalier Universitaire de Sherbrooke
4, Centre Hospitalier Universite Laval

5, Children's Hospital of Eastern Ontario
6, Dr. Everett Chalmers Regional
7, Foothills Hospital (U of Calgary)
8, IWK Health Centre
9, Janeway Children's Health & Rehab Centre
10, Jewish General Hospital
11, Kingston General
12, London Health Sciences Centre
13, McGill University Health Centre-MCH
14, McMaster University Hospital (HHS) Hamilton Health Sciences
15, Moncton Hospital
16, Mount Sinai Hospital
17, Regina General Hospital
18, Royal Alexandra Hospital & University of Alberta Hospital
19, Royal Columbian
20, Royal University Hospital/U of Saskatchewan
21, Royal Victoria Hospital
22, St. Boniface Hospital
23, St. John Regional
24, Stollery Children's Health Centre
25, Sunnybrook Health Sciences Centre
26, Surrey Memorial Hospital
27, The Hospital for Sick Children
28, Université de Montreal Hospital Sainte-Justine
29, Victoria General
30, Windsor Regional Hospital
31, Winnipeg Health Sciences Centre
33, The Ottawa Hospital
32, Other (please specify below)

IF 32:
Please specify the NICU that you work in: [Text box]

What is your professional role?
1, Registered nurse
2, Charge nurse
3, Medical director
4, Neonatologist
5, Neonatology fellow or resident
6, Clinical associate
7, Neonatal nurse practitioner
8, Pediatrician
9, Program director
10, Unit manager
11, Social worker
12, Pharmacist

13, Registered respiratory therapist
14, Dietician
15, Lactation consultant
16, Other

IF 16:
Please specify your role: [Textbox]

How many years of experience do you have in your current role?
1, Less than 6 months
2, 6-11 months
3, 1-2 years
4, 3-4 years
5, 5-10 years
6, 11-20 years
7, 21 years or more

How many years have you been at your current NICU (in all roles)?
1, Less than 6 months
2, 6-11 months
3, 1-2 years
4, 3-4 years
5, 5-10 years
6, 11-20 years
7, 21 years or more

IF 5:
Please specify: [Text box]

SURVEY INSTRUCTIONS

Thank you for participating in this Delphi (Round 1) survey! It contains 6 overarching categories and 5 – 13 individual items within each of those 6 categories. These categories and items represent important, potential national recommendations specifically **related to the COVID-19 pandemic in the NICU**. Although all categories and items have importance with respect to the COVID-19 pandemic in the NICU, we ask that you are deliberate in rating them, considering that not everything will be able to be included in the final national recommendations. This process is meant to narrow the list down to the categories and items of the *highest importance*.

First:

You will be asked to rate the 6 overarching categories of practice recommendations, according to your opinion on the importance of including each category in national recommendations for ongoing pandemic management and post-pandemic policies. You will rate each category on a scale from 1 (very low importance) to 5 (very high importance). You will have the chance to suggest additional categories or alternative wording of any existing category if you wish to do so.

Second:

Each category contains 5 – 13 individual items. You will rate each individual item within each category. Similarly, you will rate each item according to your opinion on the importance of including each item in national recommendations for ongoing pandemic management and post-pandemic policies. You will rate each item on a scale from 1 (very low importance) to 5 (very high importance). You will have the chance to suggest additional items or alternative wording of any existing items listed if you wish to do so.

At the end of the survey, we will ask if you wish to continue participating in the consensus-building. If you are, you will be able to submit your email address for the research team to contact you.

CATEGORIES

Please rate each of the following 6 categories of important topics related to COVID-19 in the NICU according to their **importance for discussion of inclusion in the national recommendations for ongoing pandemic management and post-pandemic policies:**

	**1** Very Low Importance	**2** Low Importance	**3** Some Importance	**4** High Importance	**5** Very High Importance
**COVID-19 Screening** (e.g., frequency, testing, screening questions, vaccination status)	☐	☐	☐	☐	☐
**Parent(s), Family Member(s), and Support Person(s) in the NICU During Pandemic** (e.g., number of people, switching, time limits, vaccination status, infection status)	☐	☐	☐	☐	☐
**Parent(s) Physical Needs and Well-being During Pandemic** (e.g., food, sleep space, healthcare, support)	☐	☐	☐	☐	☐
**Parent(s), Family Member(s), and Support Person(s) Engaging in Infant Care During Pandemic** (e.g., usual care tasks, breastfeeding support, skin-to-skin contact)	☐	☐	☐	☐	☐
**Communication & Education During Pandemic** (e.g., policies, information on infection risk to make informed decisions, communication technology equipment, use of devices in NICU, access)	☐	☐	☐	☐	☐
**Infection Control During Pandemic** (e.g., physical distancing, PPE provision, vaccination status, infection status)	☐	☐	☐	☐	☐

Would you like to suggest any additional **categories**?
1, Yes
0, No

Please indicate the other **category(s)** you would like to suggest:
[Text box]

Is there an alternative wording that you believe would be more appropriate for any of the existing **categories**?
1, Yes
0, No

Please indicate the changes you would like to suggest:
[Text box]

ITEMS, Category of 'COVID-19 Screening During Pandemic'

Please rate each of the following items of potential national recommendations related to COVID-19 in the NICU falling under the category of 'COVID-19 Screening' according to their **importance for discussion of inclusion in the national recommendations for ongoing pandemic management and post-pandemic policies.**

**Items**	**1** Very Low Importance	**2** Low Importance	**3** Some Importance	**4** High Importance	**5** Very High Importance
The same screening regulations as healthcare providers	☐	☐	☐	☐	☐
To enter hospital	☐	☐	☐	☐	☐
To enter NICU	☐	☐	☐	☐	☐
Rapid testing	☐	☐	☐	☐	☐
PCR testing	☐	☐	☐	☐	☐
Questions (fever, cough, difficulty breathing, contacts, travel history)	☐	☐	☐	☐	☐
Measurement of temperature	☐	☐	☐	☐	☐
Frequency of testing	☐	☐	☐	☐	☐
Vaccination status	☐	☐	☐	☐	☐

Would you like to suggest any other **items** within this category?
1, Yes
0, No

Please indicate the other **item(s)** you would like to suggest:
[Text box]

Is there an alternative wording that you believe would be more appropriate for any of the existing **items**?
1, Yes
0, No

Please indicate the changes you would like to suggest:
[Text box]

ITEMS, Category of 'Parent(s), Family Member(s), and Support Person(s) in the NICU During Pandemic'

Please rate each of the following items of potential national recommendations related to COVID-19 in the NICU falling under the category of 'Parent(s), Family Member(s), and Support Person(s) in the NICU' according to their **importance for discussion of inclusion in the national recommendations for ongoing pandemic management and post-pandemic policies.**

**Items**	**1** Very Low Importance	**2** Low Importance	**3** Some Importance	**4** High Importance	**5** Very High Importance
Define who falls into groups of: parent/guardian/family/support person	☐	☐	☐	☐	☐
Number of infant parents able to be with the infant in the NICU at the same time	☐	☐	☐	☐	☐
Number of infant (non-parent) primary care provider/designated guardians able to be with the infant in the NICU	☐	☐	☐	☐	☐
Number of infant parent support persons able to be with the infant in the NICU	☐	☐	☐	☐	☐
Number of infant siblings able to be with the infant in the NICU	☐	☐	☐	☐	☐
Switching of each person (across different days, within the same day)	☐	☐	☐	☐	☐
Leaving and returning to hospital NICU	☐	☐	☐	☐	☐
Amount of time each person can be present in the NICU	☐	☐	☐	☐	☐
Exemptions from NICU presence policies	☐	☐	☐	☐	☐
Vaccination status of parents/family members/support people	☐	☐	☐	☐	☐
Positive COVID-19 cases of parents/family members/support people	☐	☐	☐	☐	☐

Would you like to suggest any other **items** within this category?
1, Yes
0, No

Please indicate the other **item(s)** you would like to suggest:
[Text box]

Is there an alternative wording that you believe would be more appropriate for any of the existing **items**?
1, Yes
0, No

Please indicate the changes you would like to suggest:
[Text box]

ITEMS, Category of 'Parent(s) Physical Needs and Well-being During Pandemic'

Please rate each of the following items of potential national recommendations related to COVID-19 in the NICU falling under the category of 'Parent(s) Physical Needs and Well-being' according to their **importance for discussion of inclusion in the national recommendations for ongoing pandemic management and post-pandemic policies.**

	**1** Very Low Importance	**2** Low Importance	**3** Some Importance	**4** High Importance	**5** Very High Importance
Provision of food for free	☐	☐	☐	☐	☐
Provision of food for a reduced fee	☐	☐	☐	☐	☐
Access to food 24/7	☐	☐	☐	☐	☐
Access to dietary- or cultural-specific food	☐	☐	☐	☐	☐
Access to allocated space to eat and drink (in family lounge, in staff cafeteria, at infants' bedside)	☐	☐	☐	☐	☐
Provision of allocated space to sleep (number of people, location of space parent/family/support people,)	☐	☐	☐	☐	☐
Access to toilet/shower facilities	☐	☐	☐	☐	☐
Access to allocated space for use of tobacco, cannabis, or vape products	☐	☐	☐	☐	☐
Access to allocated space for cultural/religious ceremony or prayer	☐	☐	☐	☐	☐
Access to quiet parent/family space away from infant bedside	☐	☐	☐	☐	☐
Access to parent/family member(s) medication (writing and/or filling prescriptions) & healthcare	☐	☐	☐	☐	☐
Access to therapy services and psychological and emotional supports	☐	☐	☐	☐	☐
Access to parent/peer-to-peer support	☐	☐	☐	☐	☐

Would you like to suggest any other **items** within this category?
1, Yes
0, No

Please indicate the other **item(s)** you would like to suggest:
[Text box]

Is there an alternative wording that you believe would be more appropriate for any of the existing **items**?
1, Yes
0, No

Please indicate the changes you would like to suggest:
[Text box]

ITEMS, Category of 'Parent(s), Family Member(s), and Support Person(s) Engaging in Infant Care During Pandemic'

Please rate each of the following items of potential national recommendations related to COVID-19 in the NICU falling under the category of 'Parent(s), Family Member(s), and Support Person(s) Engaging in Infant Care' according to their **importance for discussion of inclusion in the national recommendations for ongoing pandemic management and post-pandemic policies.**

In terms of differing from regular practice (pre-pandemic)

	**1** Very Low Importance	**2** Low Importance	**3** Some Importance	**4** High Importance	**5** Very High Importance
Access for parents to participate in usual care tasks for their infant	☐	☐	☐	☐	☐
Access for mothers to breastfeed/access to lactation support and breast pump (in-hospital/NICU and on discharge home)	☐	☐	☐	☐	☐
Access for parents to provide skin-to-skin contact (as often as they want or for a specific amount of time (in hours))	☐	☐	☐	☐	☐
Access for family members/support people to provide skin-to-skin contact	☐	☐	☐	☐	☐
Access to touch infant in the incubator/cot	☐	☐	☐	☐	☐

Would you like to suggest any other **items** within this category?
1, Yes
0, No

Please indicate the other **item(s)** you would like to suggest:
[Text box]

Is there an alternative wording that you believe would be more appropriate for any of the existing **items**?
1, Yes
0, No

Please indicate the changes you would like to suggest:
[Text box]

ITEMS, Category of ‘Communication and Education During Pandemic’

Please rate <u>each</u> of the following items of potential national recommendations related to COVID-19 in the NICU falling under the category of ‘Communication and Education’ according to their **importance for discussion of inclusion in the national recommendations for ongoing pandemic management and post-pandemic policies.**

	**1**<br>Very Low Importance	**2**<br>Low Importance	**3**<br>Some Importance	**4**<br>High Importance	**5**<br>Very High Importance
Access to current policy procedure, recent policy updates, and explanation of policy necessity for parent(s)	☐	☐	☐	☐	☐
Provision of information on infection risk (including risk-reducing behaviours, safe use of PPE) to make informed decisions for safety in the NICU	☐	☐	☐	☐	☐
Provision of hospital equipment/technology for virtual communication in the NICU	☐	☐	☐	☐	☐
Provision of hospital equipment/technology for virtual communication outside the NICU	☐	☐	☐	☐	☐
Use of parents’ own technology devices (e.g., phone, tablet, etc.) in the NICU	☐	☐	☐	☐	☐
Training for virtual technology communication	☐	☐	☐	☐	☐
Access and support to participate in daily medical rounds (virtually, in-person at bedside, in-person away from bedside)	☐	☐	☐	☐	☐
Greater number of supports/support staff to account for increased updates and supports for parent(s)/family member(s) during periods of more restrictive pandemic/outbreak(s) policy	☐	☐	☐	☐	☐

Would you like to suggest any other **items** within this category?
1, Yes
0, No

Please indicate the other **item(s)** you would like to suggest:

[Text box]

Is there an alternative wording that you believe would be more appropriate for any of the existing **items**?
1, Yes
0, No

Please indicate the changes you would like to suggest:
[Text box]

ITEMS, Category of 'Infection Control During Pandemic'

Please rate each of the following items of potential national recommendations related to COVID-19 in the NICU falling under the category of 'Infection Control' according to their **importance for discussion of inclusion in the national recommendations for ongoing pandemic management and post-pandemic policies.**

	**1** Very Low Importance	**2** Low Importance	**3** Some Importance	**4** High Importance	**5** Very High Importance
Physical distancing procedures	☐	☐	☐	☐	☐
Open bay vs single room	☐	☐	☐	☐	☐
Provision of PPE (i.e., face masks) and hand sanitizer	☐	☐	☐	☐	☐
Instruction on expectation of wearing a face mask (upon admission to hospital, in NICU always, only when not physically distancing, etc.)	☐	☐	☐	☐	☐
Inclusion of parent partners in designing infection control/pandemic response planning/parent-related NICU policies	☐	☐	☐	☐	☐
Vaccination status of parent(s)/family member(s)/support people	☐	☐	☐	☐	☐
COVID-19 infection status of birthing person	☐	☐	☐	☐	☐
COVID-19 infection status of each parent/family member/support person	☐	☐	☐	☐	☐

Would you like to suggest any other **items** within this category?
1, Yes
0, No

Please indicate the other **item(s)** you would like to suggest:
[Text box]

Is there an alternative wording that you believe would be more appropriate for any of the existing **items**?
1, Yes
0, No

Please indicate the changes you would like to suggest:
[Text box]

Thank you!

Thank you for your participation in this study

Are you interested in participating in any of the next steps of building national recommendations?

They include:

1. Ranking the top 20 items discovered by this survey, using the same format as this survey
2. Participating in meetings to determine the final national recommendations with a group of stakeholders

Please provide your email address:
[Text box]

If you would like to communicate with the study team, please contact:
Dr. Marsha Campbell-Yeo at marsha.campbell-yeo@dal.ca.

Funded by CIHR Operating Grant - Emerging COVID-19 Research Gaps and Priorities Funding.

PRESENCE STUDY
Keeping Families Together in NICU

Restricted Family Presence in the NICU during the COVID–19 Pandemic:
Understanding impact and co–created response

Thank you for your interest.
